# The Use of Helplines and Telehealth Support in Aotearoa/New Zealand During COVID-19 Pandemic Control Measures: A Mixed-Methods Study

**DOI:** 10.3389/fpsyt.2021.791209

**Published:** 2022-01-27

**Authors:** Alina Pavlova, Katrina Witt, Bonnie Scarth, Theresa Fleming, Denise Kingi-Uluave, Vartika Sharma, Sarah Hetrick, Sarah Fortune

**Affiliations:** ^1^Faculty of Medicine and Health Science, Department of Psychological Medicine, University of Auckland, Auckland, New Zealand; ^2^Orygen, Parkville, VIC, Australia; ^3^Centre for Youth Mental Health, The University of Melbourne, Parkville, VIC, Australia; ^4^Faculty of Health, Te Herenga Waka—Victoria University of Wellington, Wellington, New Zealand; ^5^Le Va, Auckland, New Zealand; ^6^Department of Social and Community Health, School of Population Health, University of Auckland, Auckland, New Zealand

**Keywords:** telehealth, helplines, public health campaigns, pandemics, COVID-19, health services use, disease control

## Abstract

**Background:**

Early evidence suggests that the COVID-19 pandemic and associated interventions have affected mental well-being and associated health service use.

**Aims:**

the aim of this study was to examine the effect of the COVID-19 pandemic and associated public health measures on helpline and telehealth service demand.

**Methods:**

the study utilized a mixed methods research design. Segmented regression analyses were used first to identify changes in patterns of demand for Aotearoa/New Zealand national helplines (*n* = 11) from January 2020 until the end of March 2021. Thematic analysis of 23 in-depth interviews was used next to explore the reasons behind the quantitative findings from the perspective of various organizational stakeholders.

**Results:**

the data from 1,244,293 Aotearoa/New Zealand national helplines' contacts between January 2020 and March 2021 showed a non-significant (1.4%) upward trend for the full range of observations. Throughout this period, a peak and trough pattern was observed. Significant demand increases were observed in anticipation of containment measures (12.4% increase from January to March 2020) and significant demand decreases coincided with relaxation of restrictions (6.9% decrease from April to June 2020). There were spikes in demand during public health interventions (i.e., mental health promotion, introduction of new helpline services) and regional lockdowns, but these did not result in significant changes in trends. In general, the demand for helplines stabilized at a new higher level. Most of the contacts occurred by telephone calls. Contacts by other methods (webchat, text, email) have shown higher uptake during the periods of lockdowns. Quantitative-qualitative data triangulation showed that youth and populations who were disproportionally negatively affected by unstable economic conditions and underemployment made more frequent contacts. Providers emphasized that increased demand could be viewed positively as a successful outcome of public health messaging; however, greater capacity is needed to better serve higher demand.

**Conclusions:**

COVID-19, related interventions, and measures of control were associated with an increase in contacts to helplines. However, the extent of the demand increases was lower than observed internationally. Moreover, in Aotearoa/New Zealand the reasons for increases in demand were often beyond the COVID-19 pandemic and measures of control.

## Introduction

COVID-19 was declared a global pandemic by the World Health Organization (WHO) on March 11, 2020. To prevent transmission of the disease, many governments issued directives for people to stay at home, limiting travel and many work, recreational and economic activities. These measures have had a significant impact on daily life, even in Aotearoa/New Zealand which adopted a “go hard, go early” elimination strategy (see Appendices 1–3) (1). Whilst this strategy resulted in low COVID-19 infection rates and relatively few COVID-19 deaths, with New Zealanders overall experiencing much lower levels of COVID-19 bereavement and due to low infection rates, relatively fewer cases of long COVID-19 (2) when compared with most other countries (3, 4), the COVID-19 pandemic has not been without its impacts.

There was a great deal of concern that pandemic control measures would have a negative impact on mental health at a population level. Early international self-report surveys suggested increased psychological distress in the community, although there was considerable variation in the study findings and many studies had methodological shortcomings [for review see (5)]. A longitudinal study in the United Kingdom (UK) (6) found increased rates of suicidal ideation between March and May 2020, although there was no change in levels of depression or loneliness, decreased anxiety and increased well-being across the study period. An early New Zealand study of 681 respondents suggested levels of depression and anxiety exceeded pre-COVID-19 norms (7), albeit with a comparison that was based on international population data (8–10). At the same time, anxiety levels were lower amongst female participants in New Zealand compared with the UK, even though nearly half of New Zealand participants were key/essential workers, a group experiencing highly anxiety provoking situations in their work at that time (7). Another study conducted in April 2020 with 2,010 respondents suggested that nearly one third of respondents were experiencing moderate to severe distress, although nearly two thirds had experienced some “silver lining” effect of the lockdown experience (11).

Larger New Zealand community-based studies such as the New Zealand Health Survey (NZHS) suggested a small increase in prevalence of psychological distress at a population level, rising from 7.4% in 2019/20 (*n* = 12,989) to 9.3% in 2020/21 (*n* ~ 13,000) (12). A New Zealand Health Promotion Agency funded survey in April and June 2020 noted that 10% of the 1,190 respondents reported severe anxiety or depression. In contrast, nine out of 10 people reported at least one positive experience during the lockdown, particularly Māori and Pacific respondents (13). These studies also suggested that not all members of the community were equally affected by psychological distress in the early stages of the pandemic: with higher self-reported psychological distress for females; those more vulnerable to COVID-19; those in lower socio-economic groups and those unemployed or living with young children (5, 6, 14). For New Zealand specifically, younger age (11, 13) and being at higher risk of contracting COVID-19 were important factors (7).

Two of several population-level interventions are worth considering when examining the psychosocial impact of COVID-19. First, the entire population of New Zealand were subject to strong pandemic control measures and a hard lockdown commenced in March 2020. Specifically, measures included highly restricted international and national borders, strict quaranteen measures requiring New Zealanders to stay at home except for essential purposes such as grocery shopping, acute hospital care and restricted outdoor exercise and essentail work (i.e., food supply chains and health care). All social gatherings were suspended. Social contact was limited to those within households (see Appendices 2, 3). These restrictions presented a significant set of challenges. A review by Brooks et al. (15) has suggested that infection fears, frustration, boredom, inadequate supplies, inadequate information, financial loss, and stigma are associated with increased psychological difficulties in these circumstances.

The second population level intervention to consider was an extensive psychosocial and mental well-being plan *Kia Kaha, Kia Māia, Kia Ora Aotearoa: COVID-19 Psychosocial and Mental Wellbeing Plan* (16). This plan outlined a range of policy and service level activities to support population mental health during the pandemic period. It was first published in May 2020, with further revision in December 2020. Mulitmedia campaigns (e.g., “Getting Through Together,” “Struggle Got Real?”) were rolled out to normalize psychological distress in uncertain COVID-19 times. This campaign also actively promoted an array of services, including telehealth services (17, 18). Telehealth services are defined as the delivery of health care, health education, and health information services via remote technologies (19). This approach is particularly relevant to the COVID-19 period, as pandemic control measures decreased access to face-to-face, or in-person support options (20, 21).

International reports suggest that in the early COVID-19 pandemic period, helpline and telehealth service demand increased particularly following lockdowns or spikes in infection rates (22–26), often followed by decreased demand (22–24, 26). During the first months of the pandemic (January to April 2020), some countries experienced much higher increases in demand [44% in Romania (23), 50% in the United States (25)] than others [~8% in Australia for Kids Helpline (22)]. Decreased demand was observed when COVID-19 cases stabilized and pandemic control measures relaxed, but also ranged ~3% reduction in demand for Kids helpline in Australia (22) to 24% reduction in demand for a general national mental health helpline in Romania (23). More modest fluctuations were seen during the second wave of COVID-19 infections (26). Internet searches for telehealth services worldwide also spiked in March 2020, but tailed off, albeit at a higher level, thereafter (27). In contrast to telehealth demand, face-to-face presentations to healthcare services fell (25, 28). Media reports in Aotearoa/New Zealand suggested a sharp increase in calls and texts to mental health helplines following the first national lockdown, particularly by people seeking help for the first time (29). However, robust studies quantifying the extent of changes in demand associated with COVID-19 and associated control measures are scarce (22, 25) and few have longer term data collection periods.

Within the methodological constraints outlined above, these studies suggest that nearly two-thirds of contacts to helplines at the start of the pandemic were coming from females (25, 26), although lower proportion of female callers were found in a study from India (13% females) (24) and higher proportions of females in the elderly (30) and younger populations (22) (85% and ~75% respectively). In an Australian study of younger people, the increase in demand was also higher for females (5% monthly increase from January to August 2020) than for males (4% monthly increase from September 2019 to August 2020). This is also one of the few studies that document gender-diverse populations with a continuous trend of 1% monthly increase in demand from gender-diverse Australian youth between 2017 and the COVID-19 pandemic (22) period. In the United States, there was a significant increase of 5% in telehealth contacts for those aged 18–49 years from January to March 2020 (25). In Australia, contacts from children aged 5–12 increased of 9.9% per month from February until August 2020; teens aged 13–17 years had a 3.5% monthly increase in contacts (22). Younger people appeared to have shifted mode of contact, with webchat being used increasingly during COVID-19 control measures (22).

With regards to difficulties faced by people who contacted helplines, although in the early stages of the pandemic (January to February 2020) most telehealth contacts had non-COVID-19 related queries, the proportion of COVID-19–related encounters grew over time (25). Anxieties related to quarantine measures (including loneliness), COVID-19 illness fears, worries about the economy, and concern about health of loved ones emerged as the most prominent concerns (26, 31). Contacts from people seeking help about family relationships also increased, especially during spikes in infection rates and associated control measures (22). Studies have also shown increased rates of people contacting helplines with symptoms of anxiety and depression (26, 30, 31), and an increase in crisis calls in younger populations was observed in Australia (22). Again, with an exception of two studies (22, 25), these observations should be interpreted with caution due to methodological limitations.

This current study examined patterns of contact with national telehealth services in New Zealand prior to and during the COVID-19 pandemic (January 2020 until the end of March 2021) in Aoteaora/New Zealand. Telehealth services were defined as helpline services or telehealth technology that is used in the primary care/General Practice context.

The aims of this study were to:

1. Identify changes in patterns of demand for national telehealth services before and during the COVID-19 pandemic period, with variations described in terms of gender, ethnicity, age, mode of contact and reasons for contact in a methodologically robust way;
2. Explore the reasons behind observed patterns of change from the perspectives of service providers, and the implications for future practice.

## Materials and Methods

We employed a mixed methods approach; a quantitative interrupted time series analysis examining summary statistics of de-identified helpline service users' data and a qualitative thematic analysis of interviews with helpline managers and staff, General Practitioners, and other healthcare service providers to understand the impact of COVID-19 containment measures on patterns of service usage. For the qualitative aspects of the study, we followed Consolidated Criteria for Reporting Qualitative Research (COREQ) guidelines (32). For the quantitative aspects of the study, we followed The Strengthening the Reporting of Observational Studies in Epidemiology (STROBE) guidelines (33). The study was approved by the University of Auckland Health Research Ethics Committee (AH3109).

### Participant Selection and Sampling of Organizations

Between 1 November 2020 and 30 April 2021 we purposefully sampled (by funding type, size, areas of expertise, target population age, gender, ethnicity), and approached 23 (of ~33 that exist) national helplines and support organizations (henceforth commonly referred to as helplines) and 13 General Practices across Aotearoa/New Zealand (selected by area and practice size).

**Figure 1:**
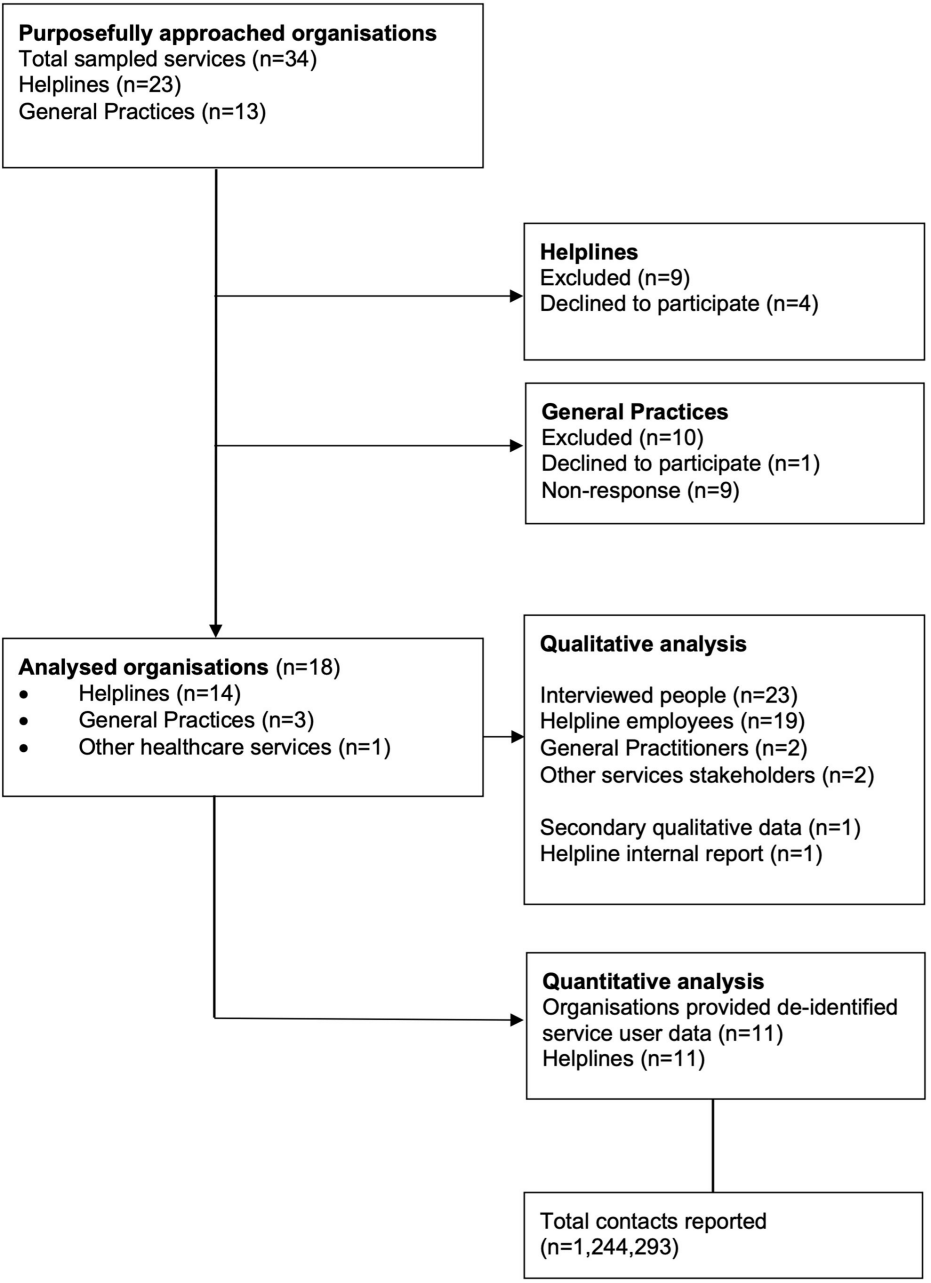
Data collection flow diagram.

Fourteen national helplines, and three General Practices agreed to participate in the qualitative interviews (see Figure 1). The final sample included national helplines representing those with public and private funding, a range of sizes (8–350+ staff/volunteers), different areas of expertise (general health, general support, general mental health, specialized services targeting presentations of anxiety, depression, substance use, eating disorders, suicide and self-harm support etc.), and various target populations such as age (general, services for youth, services for elderly), gender and sexuality (general, specific to male, specific to female, LGBTQI+), ethnicity (general, Māori, Pacific Peoples, Asian populations, people with refugee status). Some of the organizations were umbrella organizations representing multiple services. Two of the General Practices were Auckland based (Central and South Auckland) and one Christchurch based. We also included interviews with other healthcare service providers to gain insight into the contextual issues from a broader perspective.

We used a combination of snowball sampling, and cold calling to identify potential participants. Two-fifths of final participants were recruited by cold calling, with remaining three-fifths being recruited by snowball sampling. Four of the helplines and two General Practices declined to participate, and five helplines and nine General Practices never replied. Reasons for not participating were often not explained, but organizations that did participate described difficulties in finding time for the research participation due to increase service demands.

Eleven helplines agreed to provide de-identified service usage data for the quantitative component of the study. Fifty-five percent of the data provided were represented by government funded organizations and the rest by non-governmental organizations (NGOs). The data were shared by organizations of various sizes and areas of expertise.

### Procedures

The Chief Executive Officers (CEOs) of organizations were approached for consent to interview key informants and to provide de-identified service user data. Once consent was obtained, we then approached potential key informants, explaining the methodology and study aims. The key informants had to provide additional consent. In the case of non-response, we followed up with the organization once every 2 weeks to a maximum of four times. Most participants preferred for their interviews to take place on Zoom (conducted in private settings where the interview could not be overheard). Two interviews were face-to-face, two participants replied in writing. In addition, one organization shared qualitative data available from their own internal review.

We used a semi-structured interview guide (see Appendix 4) to elicit open-ended responses relevant to our research question (34). The interviewer did not previously know the participants with one exception. Interviews were audio-recorded and transcribed by a professional transcriber under a confidentiality agreement. Transcripts were de-identified by the interviewer. The audio recordings and identifiable parts of interview transcriptions were permanently deleted.

The quantitative helpline data were provided in free format and were of variable quality. Slightly more than a half of helplines provided the data in a timeseries format (cumulative weekly or monthly data). The remaining helplines provided daily de-identified service usage data that were then combined by the research team into weekly and monthly timeseries for the purpose of interrupted timeseries analyses. Nine of the helplines have been in operation during the entire study period (January 2020 to March 2021). Two of the helplines were established during the study period; one began operations in March 2020 (Week 10) and the other in May 2020, however, the data collection pertaining to the latter helpline only began in June 2020 (Week 24).

All but one organization provided mutually exclusive ethnicity and gender categories. Where multiple ethnicity data was provided, the New Zealand Ministry of Health ethnicity data protocol was used to allocate those with multiple ethnicities to one for the purposes of analyses (35). In instances where male and female genders were reported simultaneously and when people explicitly identified as transgender, a gender-diverse category was used.

### Research Team and Reflexivity

The research team consisted of eight academic researchers (including lived-experience researchers) of diverse ethnicity, currently residing in Aotearoa/New Zealand and Australia. Some researchers were trained clinicians and many held additional clinical, suicide prevention, or other advisory roles. All members of the research team identified as female. The project was funded by the Oakley Mental Health Foundation.

### Analysis

#### Quantitative Service Use Data

Segmented regression analyses were used to identify time points where a statistically significant change in trend occurred prior and during the COVID-19 pandemic in Aotearoa/New Zealand (from January 2020 until the end of March 2021). The trends for full range of observations were also considered to gain a better understanding of the overall impact independent of fluctuations. These analyses provide an estimate of the average percentage change (APC), with associated 95% confidence intervals (CIs). Although we report whether the results are statistically significant, these do not represent absolute threshold values and should not take priority in the interpretation of this work (36). Models used Poisson standard errors and the Bayesian Information Criterion (BIC) was used to guide model selection. Analyses were implemented using the Joinpoint Regression Program version 4.8.0.1 (37).

Dependent variables were: the total number of contacts, contacts per modality (e.g., telephone, text, email, webchat), contacts by repeated/unique callers (unique, repeated), contacts across gender/sex (male, female, gender diverse), age group (under 25 years old, 25–64 years old, and 65+ years old), and ethnic grouping [New Zealand European/Pākehā, Māori, Pacific Peoples, Asian, Middle East, Latin American, and African (MELAA), and “Other” ethnicities]. Weekly timeseries data were available and analyzed based on average weekly percentage change (AWPC) for the total number of contacts, contacts per type, and repeated/unique callers. Monthly timeseries data were available and analyzed based on average monthly percentage change (AMPC) for demographic data (gender/sex, age, ethnicity). All of the analyses included all of the eleven helplines, unless specified otherwise. We decided to include data from the two new helplines in our main analyses, assuming high probability of redistribution of the demand.

Missing data represented 3% of total monthly and weekly timeseries and were derived by product of weighted percentage change per period by the available count from the prior or following period, whichever was applicable.

#### Qualitative Interview Data

In this study, we used an inductive thematic approach (38) that was data driven and atheoretical. We used a general inductive approach (39), which aimed to synthesize data under the research questions, building both from a data up approach, as well as using a top down where we were examining data on the basis of the pre-established research questions. Two researchers conducted independent coding and two other members of the team looked at the data to minimize the risk of idiosyncratic interpretation before themes were discussed with the wider research group. We kept written memos with reflections on how personal beliefs and values could affect interpretation of the data, keeping in mind our duty to contribute to the improvement of national helpline services and telehealth as used in the context of primary care.

Both quantitative and qualitative results were analyzed in the context of Aotearoa/New Zealand COVID-19 pandemic related events (Appendix 1) and general (Appendix 2) and healthcare (Appendices 3A,B) restrictions associated with the official Alert Levels.

## Results

### Sample Characteristics

#### Quantitative Data

Between January 2020 and March 2021, participating helplines received a total of 1,244,293 contacts. Of these, nearly three quarters were unique, and a quarter were repeated callers. The majority of these were telephone calls, followed by text, webchat, email, and other methods (e.g., website, Facebook, other unspecified methods). Notably, for 40% of helplines telephone contact was the only service provision option. Of unique contacts, 90% have reported gender, 83% have reported age, and 75% have reported ethnicity (see Table 1). Younger people were less likely to have ethnicity and gender data available for their contacts.

**Table 1:** Sample characteristics helpline contacts January 2020 to March 2021 (*N* = 1,244,293).

**Characteristic**		**Count**
**Gender**	Male	304,575
	Female	494,664
	Gender-diverse	1,012
	Not reported	10%
**Age**	<25	293,854
	25–64	402,934
	65+	60,430
	Not reported	17%
**Ethnicity**	NZ European/Pākehā	416,525
	Māori	110,103
	Pacific Peoples	35,806
	Asian	40,647
	MELAA	5,014
	Other	56,802
	Not reported	25%
**Contact type**	Telephone	948,763
	Text	253,500
	Email	12,227
	Webchat	12,650
	Other	189
**Contact status**	Repeated	306,465
	Unique or new	934,580
	Unknown	6,222

The majority of people who contacted services were female (62%), followed by male (38%), and a smaller number were gender-diverse (<1%). In terms of age, the majority were 25–64 years old (53%), followed by people under 25 years old (39%), and people aged 65 or older (8%). In terms of ethnicity, the majority identified as New Zealand European/Pākehā (63%), followed by Māori (17%), assigned “Other” ethnicity (9%), identified as Asian (6%), Pacific Peoples (5%), and MELAA (<1%).

#### Qualitative Data

Between September 2020 and March 2021, we interviewed 23 employees/volunteers from 14 national helplines, health related services and 3 General Practices. Study participants mostly held managerial positions, but also included 35% of staff that also were in direct contact with service users. Participants were a mix of paid staff and volunteers (Table 2).

**Table 2:** Qualitative participants characteristics (*N* = 23).

**Characteristic**		**Count**
**Gender**	Male	7
	Female	16
**Age**	25–64	22
	65+	1
**Ethnicity** ^*^	NZ European/Pākehā	15
	Māori	2
	Pacific Peoples	2
	Asian	3
	Other	1
**Seniority** ^*^	Managerial role	18
	Non-managerial role	8

* *Not mutually exclusive*.

### Changes in Patterns of Demand Across the COVID-19 Pandemic Control Period Covering January 2020 to March 2021

#### General Patterns of Demand

All of the eleven helplines provided the total number of contacts per week. The timeseries analyses showed evidence of a non-significant 1.4% upward trend in contacts for the full range of observations over the full time period [95% CI −1.1 to 4.0, *t*_(63)_ = 1.1, *p* > 0.05].

**Figure 2:**
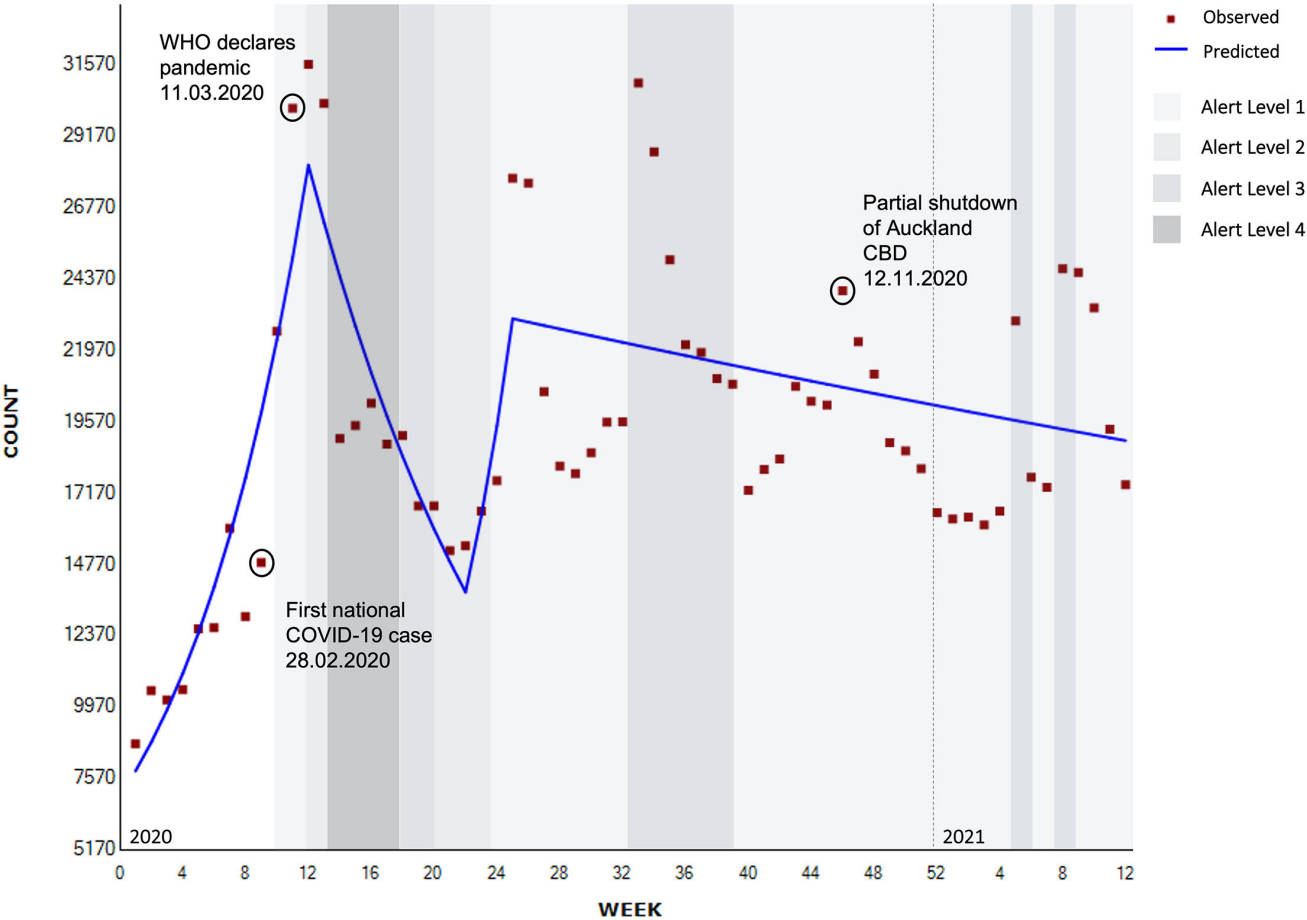
Segmented regression analysis of total contacts to national helplines (weekly) (*N* = 1,244,293).

A significant increase of 12.4% in contacts was seen between weeks 1 and 12 in 2020 (January to March 2020) when COVID-19 pandemic control measures began in Aotearoa (95% CI 8.4–16.4, *p* < 0.001). This increase was followed by a significant decrease of 6.9% from week 12 until week 22 (April to June 2020) that coincided with movement from Alert Level 4 (the most restrictive level) to Alert Level 2 (few COVID-related restrictions) (95% CI −10.7 to −2.9, *p* < 0.001) and then a non-significant rebound of 18.5% until week 25, which was associated with an introduction of a new helpline and related publicity (95% CI −28.0 to 94.9, *p* = 0.497), followed by a more gradual but significant decrease of 0.5% in total contacts (95% CI −1.0 to 0.1, *p* < 0.05; Figure 2).

Other spikes in contacts that can be seen graphically (Figure 2) rather than statistically, coincided with regional lockdowns, where Auckland (New Zealand's largest city) was partially or fully shutdown: Level 3 lockdown in August 2020 (weeks 33–35), partial shutdown of the Auckland Central Business District (CBD) (12th November, 2020, week 46), and in February 2021 with an Auckland regional Level 3 lockdown (weeks 6–10).

Participants who took part in the qualitative interviews suggested that both the COVID-19 pandemic and the associated control measures led to increased service contacts. This increased demand was interpreted as positive by some, that is normalizing contacting services for mental health support, and more cautiously by others who described it as a possible indication of elevated levels of societal distress. Participants noted that increases in demand due to lockdowns usually stabilized, with demand returning to normal levels once the lockdown measures were lifted. The greatest spike in demand was seen during the first lockdown, and less so in subsequent lockdowns.

“*In the more recent lockdowns just at level three, it hasn't been quite as profound anymore. It's been a bit more business as usual even with the lockdowns*.” (Participant 8).

Helplines, relevant healthcare organizations, and primary care providers all commented on the impact of COVID-19 pandemic on face-to-face contacts. General Practices were “…*worried that […] women that are due for their cervical smears, babies that were due for their immunisations, would be delayed*” (Participant 3). Members of the community were also thought to be “*avoiding hospitals and emergency departments*” (Participant 16). “*The sum total of the result of all of this - [as] secondary care, face-to-face services, were much more difficult to access or inaccessible - was that everybody turned to the telehealth services. So helplines' numbers just went through the roof*” (Participant 16).

Participants felt that some healthcare was likely deferred until lockdown measures were lifted, rather than switched to accessing telehealth care in the context of primary care:

“…*I mean, how do you get an anxious little girl to sit in front of the camera and talk to you?*” (Participant 3)

#### New and Repeated Contacts

All helplines provided data regarding repeated contacts, 10 helplines provided data regarding unique or new contacts, and three helplines indicated that the repeat vs. new contact status was unknown. The total number of contacts analyses by repetition status equals 1,220,886.

For the full observation period, there was a non-significant upward trend of 1.3% of unique contacts [95% CI −1.7 to 4.4, *t*_(63)_ = 0.9, *p* > 0.05], while the number of repeated contacts remained stable [AWPC 0.0 95% CI −1.1 to 1.1, *t*_(63)_ = 0.0, *p* > 0.05]. The number of contacts where repetition was unknown showed a significant downward trend of 5.3% [95% CI −8.1 to −2.5, *t*_(63)_ = −3.6, *p* > 0.05].

**Figure 3:**
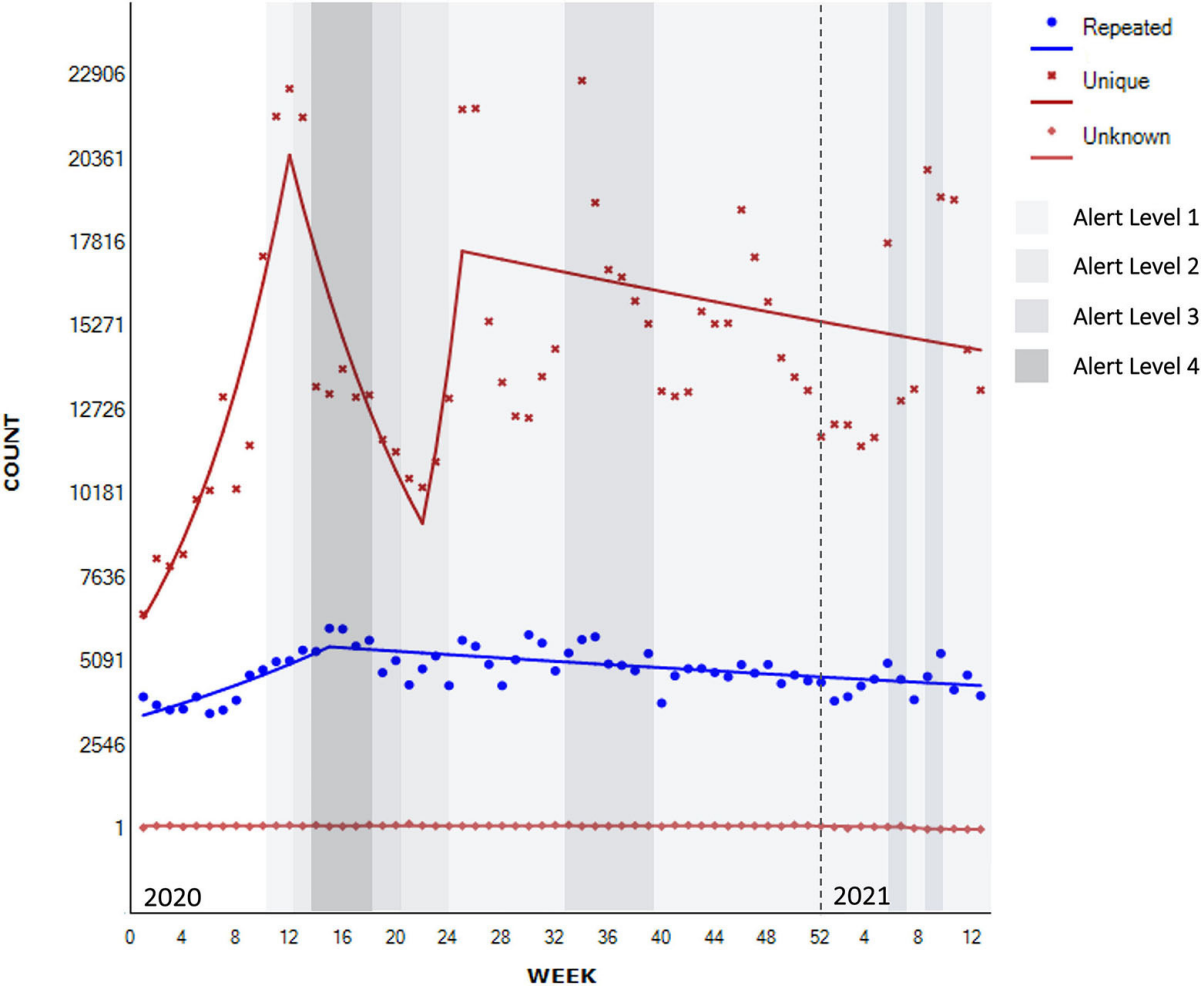
Segmented regression analysis for unique/repeated contacts to national helplines (*N*_known_ = 1,220,886).

The Figure 3 below show a significant 11.1% increase in unique (new) contacts until week 12 (January to March 2020) (95% CI 6.6–15.8, *p* < 0.001) which was followed by a significant 7.6% decrease until week 22 at the end of May 2020 (95% CI −12.1 to −2.8, *p* < 0.01), then a non-significant increase of 23.6% until week 25, or just after the lifting of the lockdown (8th of June, 2020) and when a new helpline was introduced (95% CI −32.1 to 124.9, *p* = 0.481), with a further 0.5% non-significant decrease following this (95% CI −1.0 to 0.0, *p* = 0.071).

There is also evidence of a significant 3.4% increase in contacts from repeated users until week 15 (April 2020)—leading up to and during the first Alert Level 4 lockdown (95% CI 2.1–4.7, *p* < 0.001), followed by a significant 0.5% decrease thereafter (95% CI −0.7 to −0.3, *p* < 0.001).

Interview participants also described an increase in new contacts, mainly attributed to the public health promotion campaign that normalized psychological distress in the context of the pandemic.

“*There's information out there, I think it did an amazing job of in a way normalising that people do get anxious and it's okay to reach out. So we did get some people who called us for the first time …*.” (Participant 13).

Participants also described experiencing more contact from family members, flatmates, and organizations asking about how to support their whānau (family) rather than the more typical pattern of people seeking support for themselves.

#### Contact type

**Figure 4:**
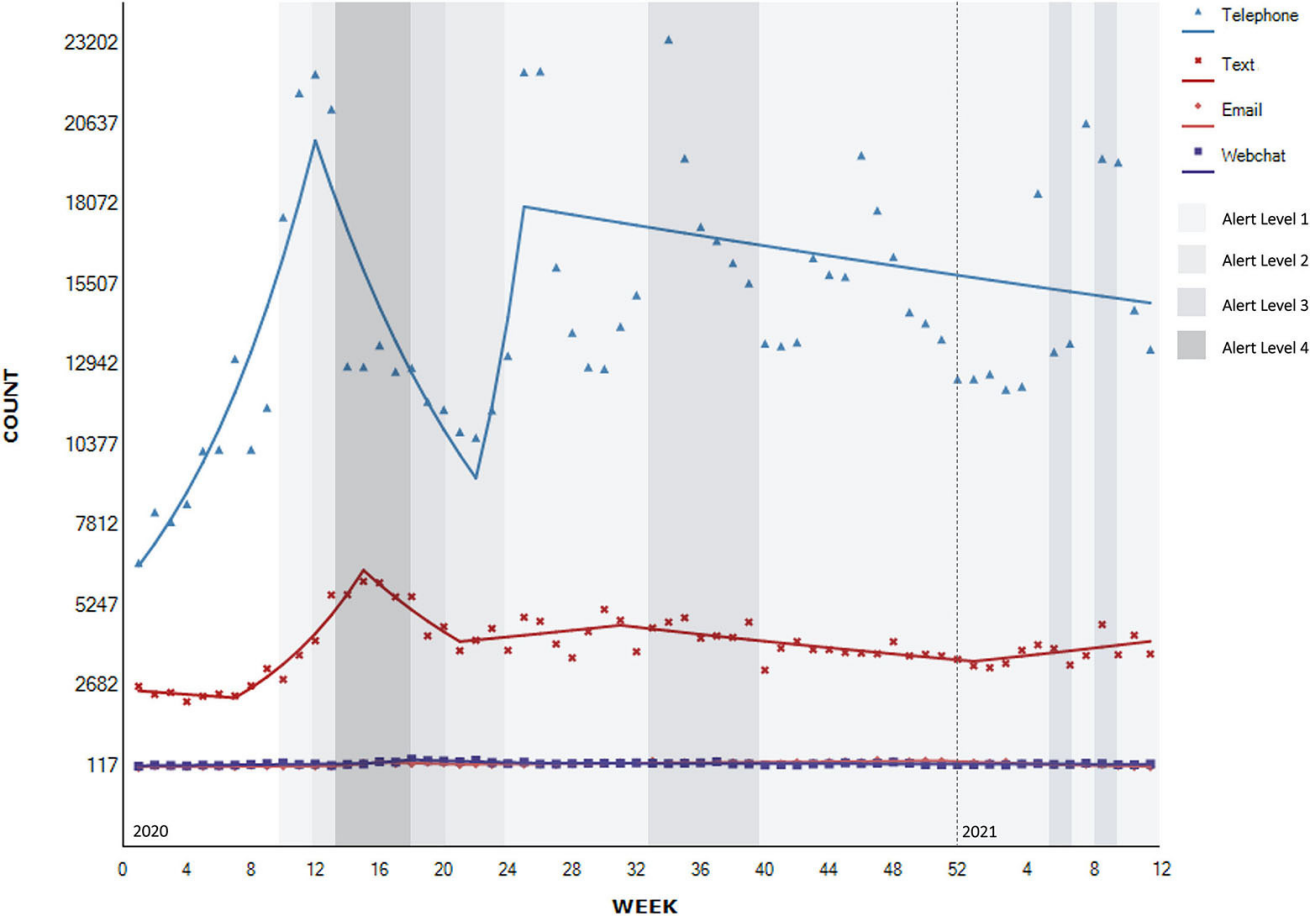
Segmented regression analysis of contacts to national helplines per contact type (*N* = 1,227,140).

Trends of contacts by contact type are provided in Figure 4. The following analyses include all telephone, text, webchat, and email contacts (*N* = 1,227,140). Not all services provided website, or Facebook channels consistently throughout the observation period, so these were excluded from subsequent analyses (*N* = 186). All of the eleven helplines provided a breakdown of contacts per contact type per week. All of the helplines had a telephone option of contact, six had an option of contact by text or webchat, and five by email. Three of the helplines could be contacted by phone only.

For the full range of observations, the timeseries analyses showed evidence of a significant upward trend of 0.8% in contacts by text [95% CI 0.1–1.5, *t*_(63)_ = 2.3, *p* > 0.05], and non-significant upward trend of 1.3% via telephone method of contact [95% CI −1.7 to 4.4, *t*_(63)_ = 0.9, *p* > 0.05] and of 0.5% by webchat method of contact [95% CI −1.2 to 2.2, *t*_(63)_ = 0.5, *p* > 0.05]. Email contacts saw a non-significant downward trend of 0.4% for the full range of observations [95% CI −2.1 to 1.4, *t*_(63)_ = −0.4, *p* > 0.05).

There is evidence of a significant 10.8% increase in telephone contacts between 2020 week 1 and week 12 (January to March 2020), in the period leading up to the lockdown measures (95% CI 6.3–15.5, *p* < 0.001), followed by a significant decrease of 7.4% during the first lockdown until week 22 at the end of May 2020 (95% CI −12.0 to −2.6, *p* < 0.01), followed by a non-significant 24.5% increase after the lifting of the lockdown to the Alert Level 1 (Week 25–8th of June, 2020) that also coincided with an introduction of an additional national helpline (95% CI −31.3 to 125.6, *p* = 0.494). This increase was followed by a more gradual non-significant decrease of 0.5% after this time (95% CI −1.0 to 0.0, *p* = 0.069).

There is evidence of a significant increase of 13.6% in text contacts from February up until week 15, or early April 2020 coinciding with the beginning of the first lockdown under Alert Level 4 (95% CI 10.9–6.3, *p* < 0.001). It was followed by a significant decrease of 7.1% until week 21 or until the easing of the lockdown to the Alert Level 2 in May 2020 (95% CI −10.3 to −3.8, *p* < 0.001), and a moderate significant decline of 1.3% from August until the end of the year (95% CI −1.7 to −0.8, *p* < 0.001) followed by a moderate significant increase of 1.5% in the beginning of 2021 (95% CI 0.3–2.8, *p* < 0.05).

There is evidence of a non-significant increase of 1.8% in email contacts between week 1 and week 13 2020 (January to March 2020) (95% CI −0.3 to 4.0, *p* = 0.088) and non-significant increase of 25.9% between weeks 13 and 16 at the start of the Alert Level 4 lockdown (95% CI −3.3 to 63.9, *p* = 0.086), followed by non-significant 6.2% decrease until week 21 2020 easing into Alert Level 2 lockdown (95% CI −13.5 to 1.7, *p* = 0.118). These fluctuations in contacts by email were followed by a moderate significant increase of 1.6% in contacts by email until week 50 2020 or mid-December (95% CI 1.2–2.0, *p* < 0.001), followed by a significant decrease of 5.2% until the end of February 2021 (95% CI −7.1 to −3.3, *p* < 0.001) and even greater significant 20.2% decrease in March 2021 (95% CI −33.2 to −4.5, *p* < 0.05).

There is evidence of a significant 3.0% increase in contacts by webchat up until week 15 (January to the beginning of April 2020) (95% CI 0.9–5.1, *p* < 0.05), followed by a further non-significant increase of 17.3% until week 18 (95% CI −16.0 to 63.9, *p* = 0.342), then a significant decrease of 5.2% to week 26 in June 2020 after transition to Alert Level 1 (95% CI −9.1 to −1.0, *p* < 0.05), followed by a significant decrease of 0.5% after this time (95% CI −0.8 to −0.1, *p* < 0 .05).

During the qualitative interviews, provider participants reflected on the changes in methods of contact (e.g., calls, texts, webchat) with observations mainly consistent with the observed quantitative data. Across helplines there was variability, with some noticing more calls, and others more texts, webchat and social media use, the latter particularly associated with younger service users. Interestingly, participants did not mention increases in emails, although this is evident in the quantitative data.

General Practitioners described an increase in the use of video consultations during lockdowns, but also noted that it did not last and was not necessarily that widespread across the country “*it was variable in terms of the level of system support and infrastructure by the organisation to support the staff and the patients to engage in a virtual consult […..] around 90% of all of the virtual consults during the lockdown period were by telephone, they were not by an audio-visual, not by AV, or video connections […] everybody has this perception that everybody was Zooming…*” (Participant 5)

Many provider participants described increased duration of contacts with service users going into more depth about their difficulties or contacting services more often. Participants felt that service users wanted to talk about general topics such as life and politics, rather than just health related topics. Participants suggested that social isolation and loneliness were behind the changes in call types to longer, more frequent calls on general non-distress related topics particularly for older people and mental health users unable to access social support. Some described an increase in prank calls.

“*The volunteers certainly felt […] that some of the conversations did go deeper, […]calls got longer. So I can't remember the numbers off the top of my head, but a lot of, a lot more calls were over ten minutes and a lot more calls were over 30 minutes than [in] the prior months*.” (Participant 2)

“*We had longer conversations with people, you know, with clients, you know more varied constant issues. Less about why they with our service [..], more about their life, more about their kids*” (Participant 6)

“*They want to talk about their political views for an hour or more*” (Participant 16).

#### Age

**Figure 5:**
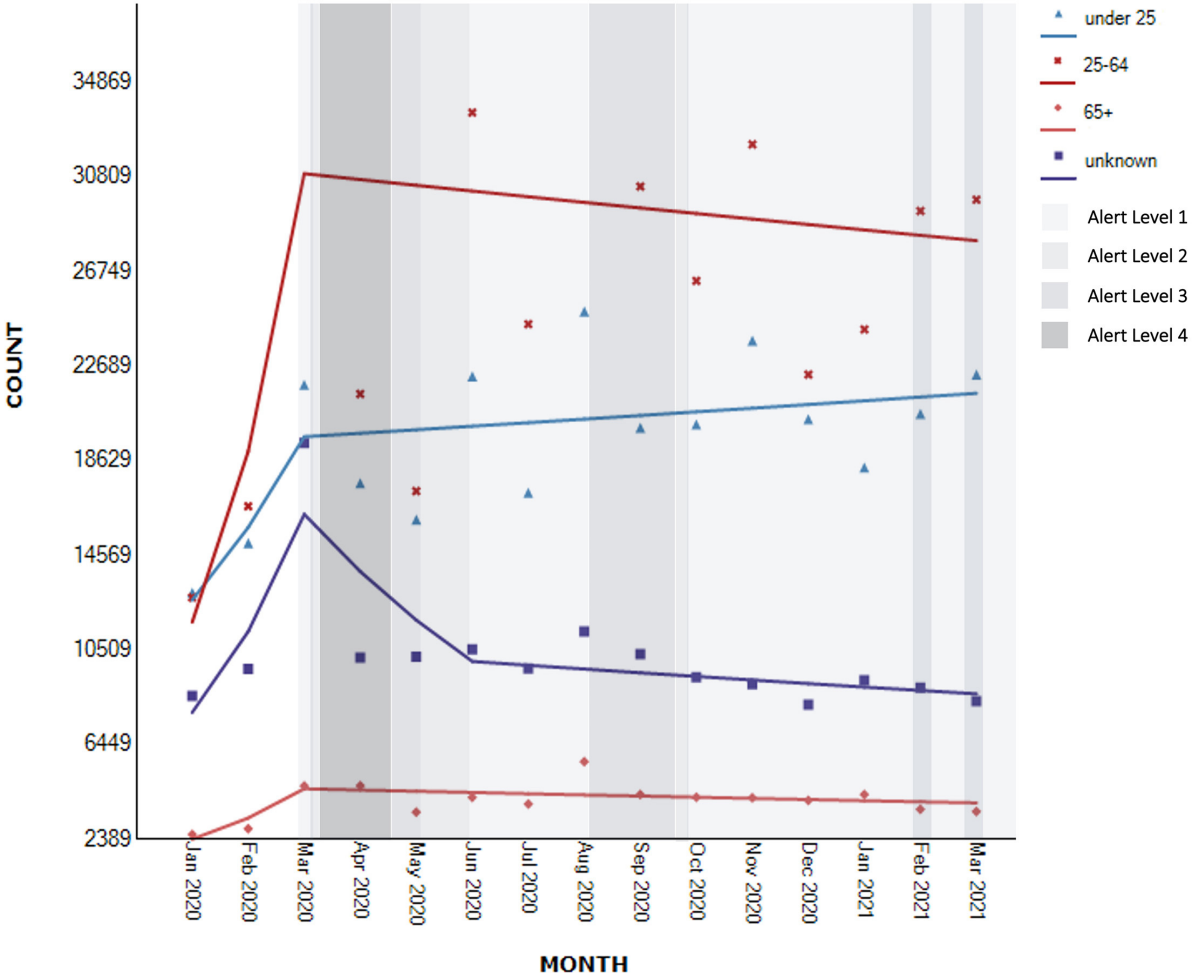
Segmented regression analysis of contacts to national helplines per age group (*N*_known_ = 757,218).

All of the helplines provided monthly information the age of service users. For one helpline only the contacts under 25 years old were flagged, while the other contacts age remained unknown. The demographic data including age (*N* = 757,218), gender (*N* = 800,251), and ethnicity (*N* = 664,897) were analysed by month. Overall, there was evidence of a change in number of contacts, per month, by age group (Figure 5).

Specifically, for the full range of observations, for young people up to 25 years, the timeseries analyses showed evidence of a non-significant upward trend of 3.9% in number of contacts [95% CI −2.7 to 10.9, *t*_(14)_ = 1.1, *p* > 0.05]. Within this trend, the analyses showed evidence of a non-significant increase of 24.7% in the number of contacts between January to March 2020 (95% CI −24.3 to 105.3, *p* = 0.348), followed by a continued slower non-significant increase of 0.8% after this time (95% CI −1.7 to 3.3, *p* = 0.515).

A similar pattern was observed for those aged 25 to 64 years and 65 years or older. There was evidence of a non-significant upward trend of 6.4% for the full range of monthly observations [95% CI −7.7 to 22.7, *t*_(14)_ = 1.0, *p* > 0.05] for those aged 25–64 years and 3.6% [95% CI −3.4 to 11.2, *t*_(14)_ = 0.9, *p* > 0.05] for people aged 65 years or older. The analyses further showed that within these full period trends, there was a non-significant increase in demand of 62.2% between January and March 2020 (95% CI −45.5 to 385.5, *p* = 0.345) for people who were 25–64 years old, and 38.0% for those aged 65 years and older (95% CI −19.4 to 136.4, *p* = 0.211). For those aged 25 years and older, this initial increase was followed by a slight non-significant decrease from April 2020 (25–64 years old: AMPC −0.8, 95% CI −5.5 to 4.1, *p* = 0.717; 65+ AMPC: −1.2, 95% CI −3.7 to 1.4; *p* = 0.335).

For those whose age was unknown the trajectory of the demand was similar to those aged 25 years or older. There was evidence of a non-significant upward trend of 0.7% for the full range of monthly observations [95% CI −4.3 to 6.0, *t*_(14)_ = 0.3, *p* > 0.05]. Within this trend, the analyses showed a significant increase in contacts of 44.4% between January and March 2020 (95% CI 13.4–84.0, *p* < 0.05), followed by non-significant decreases of 15% until June 2020 (95% CI −32.4 to 6.8, *p* = 0.136) and further 1.6% for the remaining period (95% CI −3.8 to 0.5, *p* = 0.114).

The interview data suggested a substantial focus on young people, highlighting higher demand from people of younger age although this is not reflected in the quantitative data. Some participants viewed older people as more resilient with younger people experiencing more precarious conditions in general.

“*People who, or 16–25, you know, suddenly - they've already been worried senseless about climate change and suddenly this has happened. This has all happened at a time when they're going through their last two years of school and trying to get into University and complete a degree. And everything is shut and they're having to do everything on their own at home and if they need space and peace. Just huge worries about what the future holds*” (Participant 16).

“*Thinking into those who have just graduated about just trying to get work. And I am seeing it, and very anecdotal but yeah, it suddenly occurred to me we're getting people ring us going I can't get a job. I can't go overseas, I don't know what to do with my life. I don't know where I'm going, you know, I'm out of, you know, the world's out of control, I'm out of control. Yeah, so I think will play out over several years now*.” (Participant 1)

Other participants highlighted that COVID-19 control measures resulted in older people, particularly those of Asian descent, contacting helplines more often than usual. This was attributed to this population experiencing more loneliness as a result of lockdown. However, in general, participants believed that older people might have had less reasons to worry—“*(they had) more secure sources of income, their jobs were more stable than the young ones and their casual contract*s” (Participant 3).

One participant noted that younger people had greater difficulty adjusting to the loss of freedoms. Participants also suggested that those in their middle years were having difficulty combining work from home and home-schooling children in the family.

#### Gender

Only eight helplines collected information about contacts' gender and only seven included gender-diverse category in their reporting.

There was evidence of non-significant upward trends in the number of contacts for all genders for the full observation range [males: AMPC 5.3, 95% CI −5.5 to 17.3, *t*_(14)_ = 0.9, *p* >0 .05; females: AMPC 5.1, 95% CI −4.2 to 15.3, *t*_(14)_ = 1.1, *p* > 0.05; gender-diverse AMPC 1.7, 95% CI −13.0 to 18.9, *t*_(14)_ = 0.2, *p* > 0.05].

**Figure 6:**
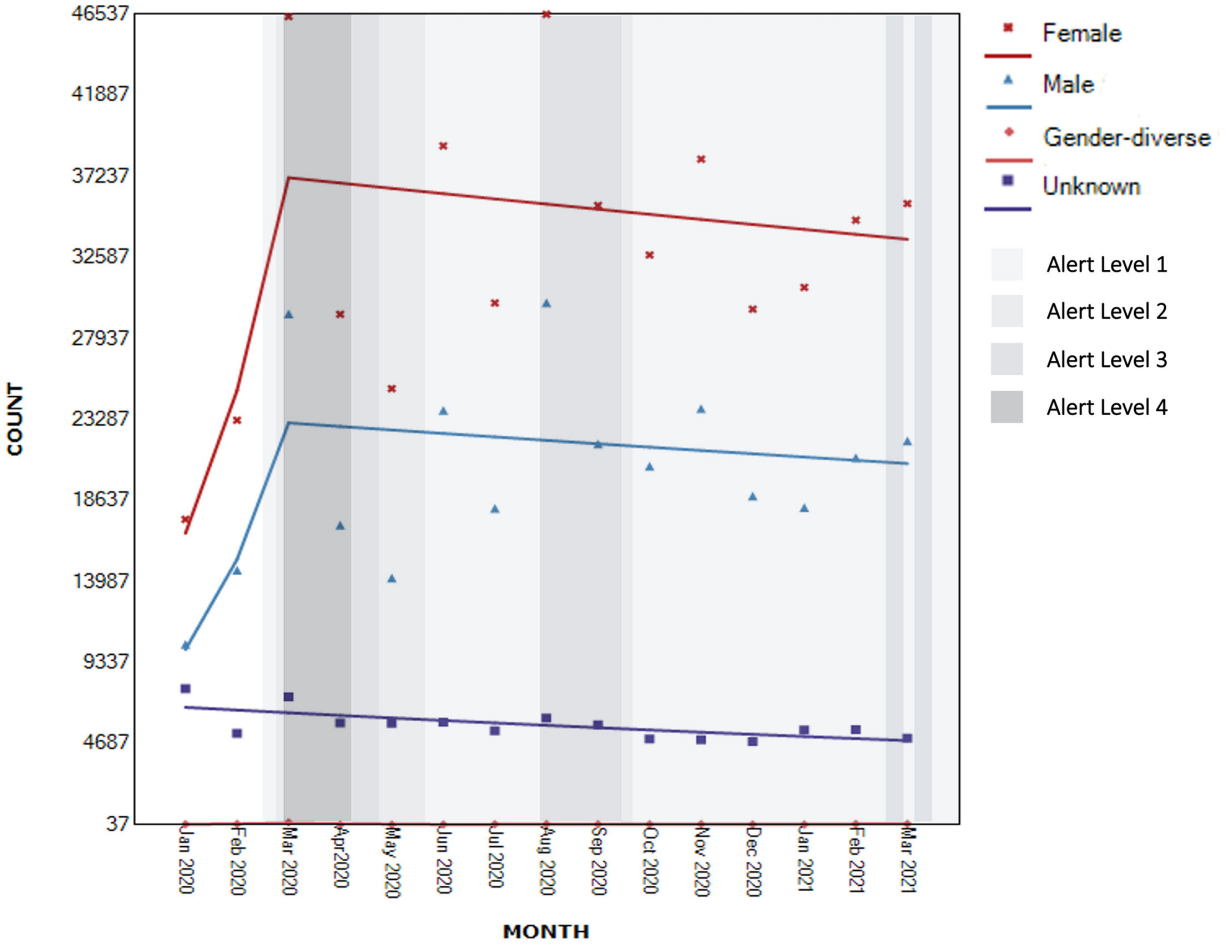
Segmented regression analysis of contacts to national helplines by gender (*N*_known_ = 800,251).

As can be visually observed in Figure 6, within these trends, there was evidence of a high, albeit non-significant, increase in contacts from males between January to March 2020 (AMPC 51.3, 95% CI −33.7 to 245.3, *p* = 0.289), followed by a modest decline after this time (AMPC −0.9, 95% CI −4.6 to 3.0, *p* = 0.619). A similar pattern was observed for females (January to March 2020: AMPC 48.9, 95% CI −26.9 to 203.3, *p* = 0.240; April 2020 to March 2021: MPC −0.8, 95% CI −4.1 to 2.5, *p* = 0.588). For those reporting diverse gender identities, there was evidence of a high but non-significant increase of 51.3% from January to March 2020 (95% CI −14.4 to 167.6, *p* = 0.129) followed by a non-significant decline of 29.3% until June 2020 (95% CI −67.4 to 53.7, *p* = 0.327) and a gradual but non-significant increase of 5.1% thereafter (95% CI −0.2 to 10.8, *p* = 0.058). The number of people who did not report their gender declined significantly by 2.3% for the entire period of observation [95% CI −3.7 to −1.0, *t*_(14)_ = −3.7, *p* > 0.05].

There were suggestions by some interview participants that there was a change in the difficulties males called for help with, including being victims of family violence and fathers contacting helpline services to enquire about how to support their childrens' well-being.

The interviewees also suggested that, for some, subject to pandemic control measures triggered personal reflections, such as reflections about one's relationships, own gender-identity and sexuality:

“*People just having a bit more time, some stuff around identity, maybe they'd kind of been pushing down or ignoring. But suddenly you're just stuck there with lots of time to think and say “well, actually…” So I think some of that definitely did come up for people*.” (Participant 2).

#### Ethnicity

**Figure 7:**
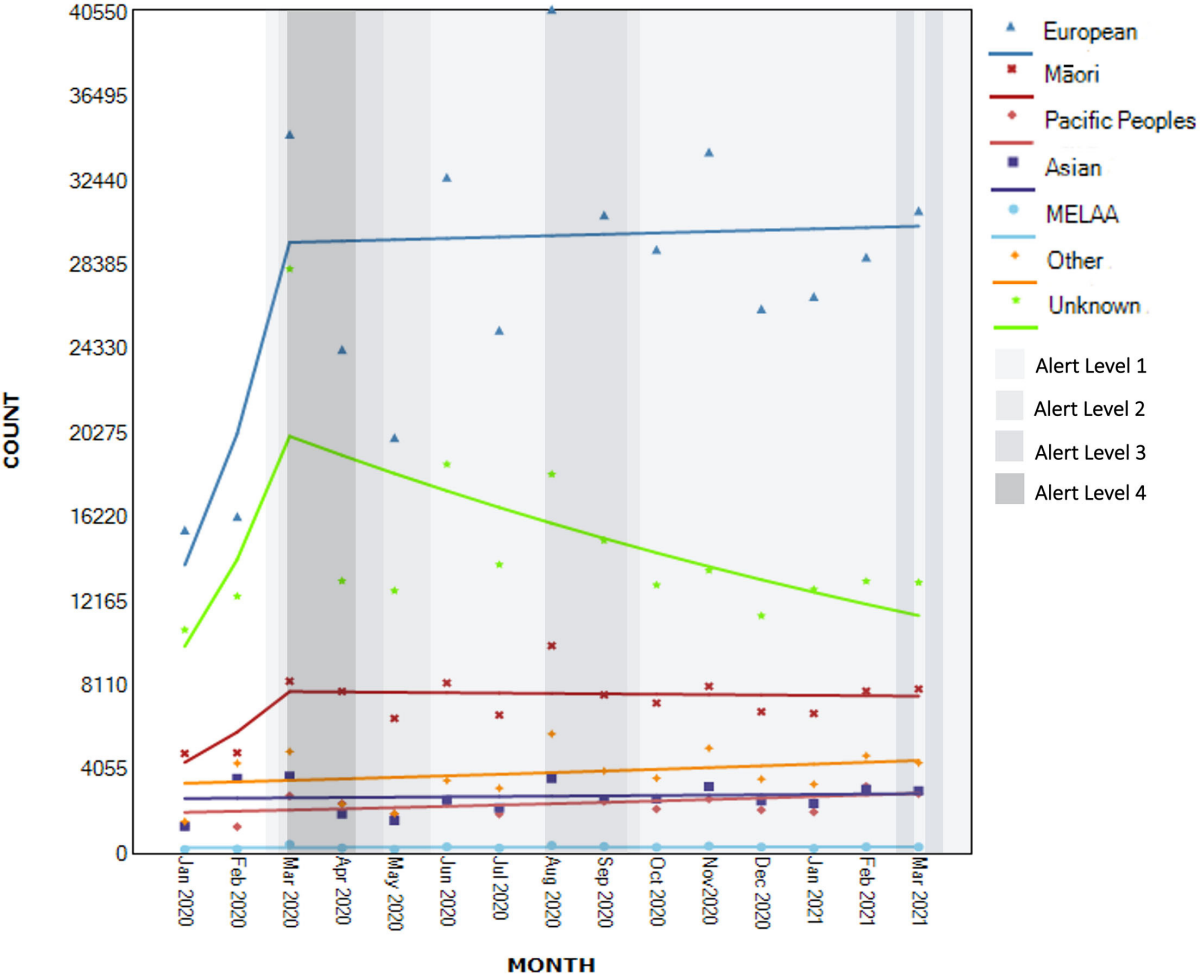
Segmented regression analysis of contacts to national helplines by ethnicity (*N*_known_ = 664,897).

Only seven helplines collected information about ethnicity. Of those, only six included MELAA as a category. There was evidence of non-significant upward trends for all ethnicities for the entire period of observation [New Zealand European/Pākehā: AMPC 5.7, 95% CI −4.7 to 17.2, *t*_(14)_ = 1.0, *p* > 0.05; Māori: AMPC 4.0, 95% CI −2.8 to 11.2, *t*_(14)_ = 1.1, *p* > 0.05; Pacific Peoples AMPC 2.7, 95% CI −0.7 to 6.2, *t*_(14)_ = 1.7, *p* > 0.05; Asian AMPC 0.6, 95% CI −2.9 to 4.2, *t*_(14)_ = 0.4, *p* > 0.05; MELAA AMPC 0.9, 95% CI −1.9 to 3.8, *t*_(14)_ = 0.7, *p* > 0.05; Other AMPC 2.0 95% CI −2.0 to 6.2, *t*_(14)_ = 1.1, *p* > 0.05– see Figure 7].

Within these trends, there was a moderate non-significant increase in contacts by New Zealand European/Pākehā between January and March 2020 (AMPC 45.4, 95% CI −34.3 to 221.8, *p* = 0.318), followed by a more modest non-significant increase after this time (AMPC 0.2, 95% CI −3.3 to 3.9, *p* = 0.895). A similar non-significant moderate increase from January to March 2020 was observed for Māori (AMPC 33.1, 95% CI −20.5 to 122.9, *p* = 0.244), but followed by a small non-significant decline (AMPC −0.2, 95% CI −2.6 to 2.2, *p* = 0.837).

For those whose ethnicity was missing, there was evidence of a non-significant increase in contacts between January and March 2020 (AMPC 41.8, 95% CI −16.3 to 140.2, *p* = 0.171) followed by a significant decrease in contacts after this time point (AMPC −4.6, 95% CI −7.3 to −1.8, *p* < 0.05).

Segmented regression analyses for contacts from Pacific Peoples, Asian, MELAA and people identifying with “Other” ethnicities did not identify additional joint points and is equal to trends described for the full range of observations.

Participants noted an increase in contacts from members of Asian and Pacific populations particularly by services that catered specifically for these populations which is not reflected in the quantitative data. These participants suggested that a large proportion of these contacts were from recent immigrants and international students. For Pacific Peoples, “*they may have been on seasonal work*” (Participant 7). Participants noted that Chinese and Korean people experienced increased prejudice and discrimination, and that Asian small business owners were highly affected. A few participants noted an increase in family violence in Asian communities, for both men and women.

“*This guy was ringing because he usually got his socialisation at the pre-school where he would meet other parents and parents would talk about children and stuff. Now he had nobody, he didn't have access to anyone because, you know, the pre-schools had closed down*.” (Participant 7)

Asian service users were also thought by providers to be more concerned about (the implications of) COVID-19 because “*both for China and India, the COVID 19 situation back home was far worse*” (Participant 19) with immigrants and refugees experiencing anxieties about families “back home”:

“*We [in New Zealand] may have shifted from COVID, the rest of the world hasn't so that's still quite heightened in their mind so and kind of like family [overseas] passing away and they're coming with grief…*” (Participant 24).

#### Socio-Economic Status

Participants described the economic consequences of COVID-19 pandemic such as job losses, financial worries, and general uncertainty about the future as provoking increased anxiety. Participants noted that those with lower socio-economic status (SES), precarious work, living in impoverished areas or in overcrowded houses were most affected.

Interviewed participants also noted people “*calling […] because of the loss of income. But that might be one person and you have a family of eight, but only one income*” (Participant 7).

“*COVID-19 might have a devastating effect on those already known to be at risk compared to second comparatively large cohort of nearly at risk people, mainly experiencing once in their lives due to changing reality*” (Participant 21).

Participants discussed an increase in calls from people employed in certain sectors including airlines, travel, hospitality, and small-business owners. These people were calling with economic concerns that were new and a consequence of COVID-19 pandemic control measures.

#### Regional Variations in Demand

The qualitative data suggested regional variations in demand, particularly from more remote areas and areas experiencing deprivation. In answering a question about what geographic areas do participants think were the most affected, one participant noted—“*low, those are known as the Hood. You know, so low socio-economical areas (there are) probably a lot of areas across New Zealand that are like the Hood (…) You know, it's [help] slower to get to them*.” (Participant 4).

People from areas like “*Queenstown District Council, [that have been] significantly affected by the socio-economic impact of COVID*” (Participant 21) and the Auckland region due to additional lockdowns. In particular, the demand from “*Pacific people in South Auckland [*were noted as*] there were increased levels of distress*” [in the context of a cluster in this community] (Participant 5).

### Changes in Types of Concerns Related to COVID-19

#### COVID-Related Health-Related and Generalized Anxiety

Interview participants in both helplines and General Practices described a number of service users expressing anxiety about COVID-19 infection as well as general illness. People were anxious because they did not know how to behave—“*should I go visit Grandma? Shouldn't I go visit go Grandma?*” (Participant 20) and were concerned about their own family members not following the rules. Some participants noticed concerns about vaccination.

General Practitioners noted anxiety about decreased access to care, which manifested differently for different types of populations: in higher SES-area people “*were more angry that they couldn't just walk in to the practice*,” for lower SES-area practice people were questioned “*what if I die and I can't come in? What do you mean I can't come in?*” (Participant 3).

General practitioners observed higher levels of somatization (e.g., medically unexplained headaches, muscle pain, etc.).

“*They didn't ring up to say, you know, I'm feeling depressed and anxious. They had physical symptoms, not necessarily COVID related, like muscular skeletal and headaches and I found it wasn't long before you just got the anxiety. So, I'd say, you know, what are the headaches, when did they start, and all of this. A lot of these people have casual contracts with places and*, “*You know, my employer has said if we're locked down for another week, then I'll be given my notice, you know, I won't be able to…*”, *those kind of things. So obviously, a lot was playing on people's minds already, which is really interesting, eh, 'cause I thought, well, I suppose people still want to talk to their doctor about their health*.” (Participant 3)

“*I feel like I've seen more people with somatic complaints that are probably more to the more psychological medical through all of this […] I don't know whether people are just more sensitive to their inner workings at the moment*.” (Participant 8)

#### Lockdown-Related Concerns

Participants noted a potential increase in service users expressing feelings of social isolation and loneliness, especially during lockdowns.

“…*when COVID and lockdown happened it sort of, to a large extent, decreased that sense, the mobility and restricted contact. And a lot of people just felt very socially isolated. So I got a lot of calls in terms of just feeling alone and lonely*.” (Participant 20)

Some participants noted that lockdowns were challenging for their target population because of lack of space and loss of freedom. These concerns appeared to be exacerbated by difficult relationships.

“*There was one […] quite a striking example. Someone that reached out who had the day before the initial lockdown, had just broken up with their partner and suddenly wasn't able to leave […] in a very small apartment and was really struggling, was really struggling*” (Participant 9)

For some, being locked-down triggered personal reflections and the need to reconcile.

“*Some current things that are coming up is having people […] holding on to things that they hadn't confessed to their partners, that happened seven weeks, seven years ago. And they've decided that it's coming up now during COVID, and they wanted to break out and talk about it*.” (Participant 17).

Participants also described increases in family violence that were thought to stem from the lack of coping strategies:

“*People are stressed out but are not talking about it, they're bottling it up. They're coping, they have poor emotional regulation, poor coping skills. So we're just talking about that, of course it's a long process to change the mindset*” (Participant 19)

#### Demand From People With Pre-existing Mental Health Symptoms

Interview participants highlighted a perception that service users with pre-existing anxiety suffered more. “*Lockdown was a mission for people who were really anxious, [people with] obsessive compulsive disorder, anxiety disorder problems*” (Participant 16).

In addition, there was a sense that presentations reflecting serious mental illness rather than general psychological distress had increased such as “*presentations of psychosis where people are saying that, you know, seeing and hearing voices and describe them […]. That trend has been in place for several years now, but it seems to have been accelerated by the lockdown experience*” (Participant 1).

Participants also reflected on their perception of increases in crisis calls particularly for younger participants.

“*We're talking about high to moderate suicide ideation, high to moderate concerning levels of self-harm. So these are not people just telling me they've cut themselves and this is a wee scratch and has stopped bleeding. But people are describing their wounds as quite serious. We're seeing very high levels of things that we never really saw before and it is part of a growing, a continuing trend I'd say, but it seems like COVID-19 has accelerated it*.” (Participant 1).

#### Protective Effects of COVID-19 Related Measures and Feelings of Social Cohesion

Helpline workers and General Practitioners noticed a potential protective effect from COVID-19 control measures where people felt part of a community. Participants noted that among some of their regular callers who struggle with social anxiety and the business of everyday life, there was a sense of relief.

“*A lot of people had consciously decided to make changes and as to how they connected with each other and how they connected with their neighbours. A lot of people had decided to do, have less time, less TV time maybe and more connection. A lot of people had, that I've heard of, they had decided to keep their family debates, you know, they're debating things, and retained their board games, they bought the board games back on*.” (Participant 6)

Other COVID-19 pandemic control measures related improvements were seen in areas of non-domestic sexual assault, particularly for younger people, relief from gambling, alcohol use and self-harm due to the inability to access means.

### Changes Not (Directly) Related to COVID-19

Although some COVID-19 concerns were novel, participants warned against attributing any increases in demand solely to COVID-19 pandemic, and spoke about how the COVID-19 pandemic and control measures simply exacerbated problems that already existed. Participants noted that year-on-year growth in demand for helpline services has been apparent for several years.

“*I think we have to be very, very careful. ……. whilst we'd seen an uplift in the last twelve months in volumes, my belief is that is not necessarily attributable to COVID. Because it's no greater uplift than what we have seen over the last five years into the services. The growth rate actually over the last year has been less than the growth rate had been in the previous four years. And so there's actually an argument the volume is higher but the growth rate if you look back in 2019, 18, 17, […] was higher in those years than what it was last year*.” (Participant 14)

Participants also highlighted other traumatic events experienced in Aotearoa/New Zealand in 2020 including earthquakes, Christchurch terror attacks, White Island volcano eruption, elections, police shooting, political protests, and a threat of tsunami.

“*There were other things that happened too as well, like Whakaari/White Island, there was anticipation around the hearing for the Mosque shooter. So COVID wasn't the only context in which we were working*.” (Participant 12)

#### The Role of Promotion and Media

Participants attributed increases in demand to increases in health promotion, including promotion for helplines by central government. There was a sense that this was particularly true for youth, and due to social media promotion strategies “*because younger audiences, that seemed to be who the algorithm was driving the, the posts to*” (Participant 2).

#### Areas for Improvement in Healthcare Infrastructure Overall

The helpline interview participants suggested that increased demand from mental health service users and more crisis calls reflected their perception that mental health services are struggling to cope with demand. Participants felt that District Health Boards lacked surge capacity and barriers to accessing mental health treatment such as understaffed services, waitlists and strict eligibility thresholds made calling helplines easier and “*you (don't) have to yell at everybody that attracted the police to your home*” (Participant 7).

Some populations (e.g., Pacific Peoples, Asian) were perceived as being more likely to be excluded from access to general healthcare due to immigration status, financial or language problems. Remote and poorer areas were perceived as being “*the last place to get stuff*” (Participant 4), exacerbated by the fact that people in such settings are “*very reluctant to contact local services within small communities, 'cause they all know each other*” (Participant 1).

Participants raised concerns about the current healthcare infrastructure. They felt that current healthcare services are fragmented with insufficient information technology (IT) infrastructure and workforce expertise to support telehealth services infrastructure (especially in relation to video-calling).

“*The mental health services in District Health Boards were trying to figure, I mean they came from a prehistoric start point in terms of delivering care virtually. District Health Boards are the most cumbersome slow to change services that we've probably got*.” (Participant 16)

Participants acknowledged that COVID-19 pandemic sped up innovation and cooperation, potentially improving services effectiveness and accessibility. However, the sustainability of such innovations and the risks of reverting back to normal were noted:

“*If you haven't seen somebody after three days, what we found was that we actually needed to pull the staff out into the community to go and actually physically see people from the door. What we found was that a couple of people had been re-admitted back into the mental health unit. Because on the phone, they were like, okay, okay, but you didn't know that they haven't slept for three days? […] there was on guy that nearly had his leg amputated. He didn't want to tell, he felt embarrassed to tell our support worker because he felt like, you know, he hadn't looked after himself, or he hadn't done, and that was, that was somebody with mental health*.” (Participant 7).

“*In Christchurch after the earthquakes, it revolutionised the delivery of health care in Canterbury. Everything that they'd relied upon to run a health service got thrown in the air. And for two years they were able to make changes on a scale that had never been seen before all of which are fantastic. But then in about year 4, 5 and 6 they started to just to revert back, not completely, but I'm just wondering how that's going to go. I'd love to see us learning from this in everything from climate change through to normal health care. I think that it's time for [services] to deliver care in a far, far more responsible and meaningful way, it is so overdue now*.” (Participant 16).

## Discussion

The population of Aotearoa/New Zealand were subject to strong pandemic control measures, and a hard lockdown commenced in March 2020. There were concerns the COVID-19 pandemic could have a negative impact on mental health at a population level. Early self-report surveys suggested increased psychological distress in the community, although there was considerable variation in the study findings, and many studies had methodological shortcomings [for review see (5)]. In any event, the Aotearoa/New Zealand Government invested in a population level mental health intervention *Kia Kaha, Kia Māia, Kia Ora Aotearoa: COVID-19 Psychosocial and Mental Wellbeing Plan* (16), which included funding and promoting of a range of helpline and telehealth services, both new and existing.

This mixed methods study is the first of its kind internationally to have examined changes in patterns of demand for national telehealth services before and during the COVID-19 pandemic period, with variations described in terms of gender, ethnicity, age, mode of contact and reasons for contact. Between January 2020 and March 2021, New Zealand-based helplines included in this study received a total of 1,244,293 contacts of which nearly three quarters of the contacts were unique, and a quarter repeated. The majority of these were telephone calls, followed by text, webchat, email, and other methods. Nearly two thirds of those who contacted services were female (62%) and more than half were 25–64 years old. Two in five contacts were from people under the age of 25 years and only a small proportion were over 65 years. The demographic representation is similar to such observed in other international studies (25, 26). The majority of those who contacted services were of New Zealand European/Pākehā ethnicity (63%), followed by Māori (17%), those who identified as ‘other' ethnicity (9%), Asian (6%) and Pacific Peoples (5%).

There was a significant 12.4% increase in contacts between January 2020 and March 2020 when the COVID-19 pandemic was declared and New Zealand went into a hard lockdown in late March. Further spikes in contacts coincided with national and regional lockdowns and the introduction of a new helpline in mid 2020. Our findings are consistent with international studies showing helpline service demand increased following lockdowns or spikes in infection rates (22–26), followed by decreased demand (22–24, 26). Moreover, although there was a gradual 0.5% decline in contacts after the first national lockdown, and we have seen some peaks and troughs during regional lockdowns, the overall demand stabilised at a new, higher level with an overall upward trend. This indication of a new higher level of demand based on longitudinal data is a novel finding, although the gradual increase in demand throughout the study period did not reach significance levels. We note that the increases and decreases in demand were more consistent with observations in Australia (~8% and 3% respectively) (22) and not the observations from the United States or Romania (~50% increase) (23, 25), albeit the Australian data was youth-related. Notably, it is important to acknowledge that qualitative data has shown that contacts to helplines had been already increasing prior to the COVID-19 pandemic control measures, similar to observations by the longitudinal Australian study (22).

*Kia Kaha, Kia Māia, Kia Ora Aotearoa: COVID-19 Psychosocial and Mental Wellbeing Plan* (16) encouraged new users to make contact with support services, and there was a 11.1% increase in new contacts January to March 2020 followed by non-significant but important increase of 23.6% following the introduction of a new helpline and extensive national health promotion in June 2020. In addition to the new contacts, there was also evidence of a significant 3.4% increase in contacts from repeat service users leading up to and at the beginning of the Alert Level 4 lockdown. Key informant interviews attributed the increase in new contacts to the mental health promotion campaign (i.e., public service announcements on national television, social media channels, public health promotions in public spaces via banners, in transport etc.) that normalized psychological distress in the context of the pandemic. Providers emphasized that increased demand could be viewed positively and as a successful outcome of public health promotion messaging. In fact, the higher rate of the demand increase, although not significant, might indicate that the promotional campaign has a delayed lifecycle. Importantly, increased contacts from first time users supports this hypothesis, however, increased contacts by regular users could also indicate additional distress within the community. Participants also reported more contacts from family members and peers asking about how to support others rather than the more typical pattern of people seeking support for themselves. A similar dynamic regarding concerns for the loved ones and an increase in family-related and relationships-related contacts was observed in Australia (22) and Greece (31).

Different patterns of demand were seen across different modes of contact; text contacts nearly doubled at the beginning of the first lockdown under Alert Level 4 with a small but significant 0.8% increase across the study period. There was an increase in contacts by email up to December 2020. The timeseries data showed that digital contacts by text, email, and webchat increased during the first lockdown under Alert Level 4, while phone contacts dropped. This suggests that some people felt contact by digital means was more private or practical during lockdown periods. Similar increases in contacts by digital methods, particularly Webchat during lockdown, have been observed in Australia among those contacting a youth helpline (22). The fact that only text contacts showed the significant upward trend throughout the study period might indicate that text is becoming a more popular method of contact in general and should be considered by telephone-only helplines.

Earlier studies suggested that younger people were more likely to be affected by the COVID-19 pandemic (7, 11, 13), and our study found that contacts by young people under 25 years increased throughout the study period, whereas contacts by people those older than 25 years gradually declined once the first lockdown lifted. In addition the number of over 65 year olds contacting services was smaller than by people under 65 years, similar to other studies (30).

Although the pattern of demand from males and females was similar—increasing leading up to the first lockdown and gradually declining thereafter—qualitative observations suggested more contact from males seeking support for other family members and as victims of family violence. Contacts for gender-diverse populations increased from January to March 2020, followed by a decline during the period of the first lockdown, and followed by another increase thereafter. We note that comparative to the Australian data (22), demand from males showed a higher percentage increase than the demand from females. Our results are similar with regards to gender-diverse population.

Among Māori, there was a non-significant increase in demand leading up to the first lockdown followed by a gradual decrease of 0.2%, in comparisons to other ethnic groups that saw an increase in demand. Māori experience systematic inequities in access to healthcare services and the observed pattern of demand may either reflect greater resilience among Māori communities or, alternatively, a lack of fit between Māori health needs and helpline and telehealth support approaches. A non-significant increase was observed by members of the community identifying with Asian, Pacific, MELAA and “Other” ethnic identities across the study period, which contrasts with the New Zealand European/Pākehā demand that had a larger, but also non-significant increase leading up to the first lockdown, and a gradual increase of 0.2% thereafter.

Service providers reported that some callers were reluctant to overload their usual service providers, or some were having difficulty accessing these (20, 21). From a qualitative perspective, providers attributed the increase in helpline demand between January 2020 and the end of March 2021 to COVID-19 related anxieties such as fears of infection, general anxiety about accessing healthcare, uncertainty about COVID-19 rules, questions about vaccination, and feelings of loss of control similar to other international studies (25, 26, 30, 31) and observations in Brooks and colleagues rapid review (2020). Although, similar to the data from Greece and Malta (26, 31), our qualitative results indicated potential increases in generalized anxiety; however, depression has not featured strongly in our data. Similar to the Australian youth helpline study (22), our qualitative data also pointed at higher crisis calls related to suicidality and self-harm in youth.

The population of Aotearoa/New Zealand were subject to strong pandemic control measures, and a hard lockdown commenced in March 2020. Qualitative data suggested that for some, the experience of lockdown was stressful due to feelings of loneliness and social isolation or, conversely, due to inability to separate from others. The former may be especially relevant for communities who hold collectivistic values (e.g., Māori, Asian, Pacific Peoples), elderly, and people with pre-existing mental health conditions. The latter was exacerbated by difficult relationships or younger age and the loss of freedom as observed in international studies (22, 30, 31). Service-providers described increased duration of contacts with service-users going into more depth about their difficulties, or contacting the service more often. Participants felt that service-users wanted to talk about general topics such as life and politics, rather than just health related topics due to social isolation or loneliness.

Helpline workers and General Practitioners noted some protective effect of the COVID-19 control measures where people felt they were a part of a community and among some of their regular users with social anxiety and the business of everyday life, there was a sense of relief. Some providers also noted there were fewer clients seeking help for non-domestic sexual assault, particularly among younger people, relief from gambling, alcohol and self-harm due to the inability to access means during the COVID-19 control measures.

Service providers warned against attributing variations in demand solely to the COVID-19 pandemic, and felt that COVID-19 exacerbated existing problems. Participants noted that year-on-year growth in demand for the helpline services has been apparent for several years. The helpline interview participants suggested that increased demand from mental health service users and more crisis calls reflected their perception that mental health services are struggling to cope with the demand. General Practitioners also indicated difficulties in accessing community mental health services for patients due to eligibility thresholds leading to disruptions in continuity of care for people with mental health difficulties.

Participants raised concerns about current healthcare infrastructure including IT and workforce expertise to support audio-visual telehealth technology. Some participants felt that COVID-19 had sped up innovation and cooperation potentially improving access to services. However, the sustainability of innovations and the risks of reverting back to traditional approaches to delivery were noted. There is also a need to robustly appraise the innovations in practice that were rolled out rapidly to ensure that the fundamentals of best practice have not been inadvertently compromised.

Although the quantitative data showed a stabilization and gradual decrease in helpline demand, it is mainly non-significant. This might be indicative of the fact that the impact of the pandemic has not yet passed, and economic consequences such as rising food and housing costs likely adversely affect the most vulnerable members of our community (40). Future COVID-19 infections, particularly the Delta strain, may have further impact, especially on populations who are already disproportionally affected. Disruption in healthcare access may be problematic and exacerbate chronic conditions or worsen prognosis (41, 42). Telehealth, although a great solution for many, might not be suitable for everyone, such as, for example, people with limited access to internet or mobile data or children or Māori who prefer kanohi ki te kanohi (face to face) consultations. More research is needed to understand the extent to which telehealth, both helpline services and telehealth in primary care, is congruent with the values of those seeking healthcare.

## Limitations

First, the study analyzed timeseries of data from January 2020 until March 2021. However, the COVID-19 pandemic and pandemic control measures continue, and we anticipate re-approaching helpline providers in 2022 for further data. Secondly, it should be noted that demographic data is not uniformly collected by helplines leading to high non-report with some data (e.g., gender) that may be based on volunteers' judgment or be misrepresented by the contacts themselves to retain anonymity (e.g., age, ethnicity). Finally, quantitative analysis of ethnicity data should be interpreted with caution as other newer targeted helplines emerged during the COVID-19 with the aim to provide better cultural support, but not all of these had data available for inclusion in this study.

## Conclusions

COVID-19 pandemic control measures were associated with an increase in contacts to helplines in Aotearoa/New Zealand. Variations in pandemic control measures resulted in a peak and trough pattern of demand, accompanied by a more general upward trend throughout the study period. Demographically, the demand from younger people saw the fastest increase. The demand increase from males was higher than from females, although females still represented nearly two thirds of all contacts. The demand from Māori saw a slow gradual decline, while the demand from other ethnicities continued to rise. More research is needed to understand how to provide targeted support for the populations who sought help from helplines more often. Additionally, whether the population who contacted helplines less often (i.e., older people, Māori) are more resilient and why, or whether there are other reasons for lower demand (i.e., lack of access, lack of cultural safety) should be explored. This will help to provide better systemic solutions.

Finally, although the reason for contacting helplines in Aotearoa/New Zealand were similar to those internationally, the extent of the demand increases was lower in Aotearoa/New Zealand possibly because of lower infection rates, COVID-19 related mortality, and bereavement at that time. Moreover, in Aotearoa/New Zealand the reasons for increases in demand were often beyond the COVID-19 pandemic. Future research to understand how other external factors such as other national events and emergencies, media communications, the state of economy, public health policy as well as the state of healthcare system in general may affect the demand for helplines and telehealth is urgently needed.

## Data Availability Statement

The datasets presented in this article are not readily available because the ethics approval restricts us from sharing organizational data. Requests to access the datasets should be directed to Alina Pavlova alina.pavlova@auckland.ac.nz.

## Ethics Statement

The studies involving human participants were reviewed and approved by the University of Auckland Health Research Ethics Committee (AH3109). All the organisations and interviewees provided their written informed consent to participate in this study.

## Author Contributions

SH and SF initiated the study. KW and AP conducted the quantitative analyses. BS and AP conducted the qualitative analyses. SH and SF supervised the drafting of the manuscript by AP. AP collected and prepared the data and interviewed the participants. All authors participated in the recruitment to the study and in active discussion in the process of writing of this study.

## Funding

This study was funded by Oakley Mental Health Foundation.

## Conflict of Interest

The authors declare that the research was conducted in the absence of any commercial or financial relationships that could be construed as a potential conflict of interest.

## Publisher's Note

All claims expressed in this article are solely those of the authors and do not necessarily represent those of their affiliated organizations, or those of the publisher, the editors and the reviewers. Any product that may be evaluated in this article, or claim that may be made by its manufacturer, is not guaranteed or endorsed by the publisher.
